# Supplementary material for: Evaluation of Colon-Specific Plasma Nanovesicles as New Markers of Colorectal Cancer
Source: Cancers (Basel). 2021 Aug 3;13(15):3905. doi: 10.3390/cancers13153905 (PMC8345452; doi:10.3390/cancers13153905)
Supplement: Supplementary file 1 [file cancers-13-03905-s001.zip › cancers-1262047-supplementary.pdf]

cancers MDPI

Evaluation of Colon-Specific Plasma Nanovesicles as New Markers of Colorectal Cancer

Inga Nazarova [1], Maria Slyusarenko [1], Elena Sidina [1], Nadezhda Nikiforova [1], Vladislav Semiglazov [1,2], Tatiana Semiglazova [1], Achim Aigner [3], Evgeny Rybakov [4], Anastasia Malek [1,5]

Supplementary Table S1. List of colon epithelium-specific protein surface markers (complete data)

Protein name	UniProt ID	Colon-specific expression [1]	Surface membrane localization [2]	Exo Carta ID	CD9				CD63			
					CRC patients	health donors	CRC vs HD	AUC	CRC patients	health donors	CRC vs HD	AUC
CLRN3	Q8NCR9	27.7	***	119467	7.71	2.8	ns	0.76	3.2	1.4	**	0.78
GPA33	Q99795	46.9	*****	10223	4.8	4.1	ns	0.54	2.1	1.5	ns	0.67
GCNT3	O95395	37.6	****	9245	6.4	2.5	ns	0.64	2.8	1.0	*	0.71
PIGY	Q3MUY2	10.6	*****	84992	7.2	3.3	ns	0.66	3.2	1.3	**	0.78
REG4	Q9BYZ8	55.5	***	83998	7.5	2.2	*	0.78	3.0	1.0	*	0.74
MEP1A	Q16819	100.5	*****	4224	8.1	2.8	*	0.79	3.6	1.5	ns	0.81
LGALS4	P56470	190.6	****	3960	5.6	2.4	ns	0.69	2.2	1.2	ns	0.71
Mucin 12	Q9UKN1	25.2	*****	-	7.3	2.7	*	0.75	3.0	1.2	*	0.78
PDCD6IP	Q8WUM4	36.8	*****	10015	5.7	4.1	ns	0.6	2.5	1.7	ns	0.66
DHRS11	Q6UWP2	38.7	**	360583	7.7	3.2	ns	0.63	2.7	1.4	ns	0.68
CD47	P16150	25.7	***	961	3.1	2.1	ns	0.51	2.0	1.5	ns	0.60
VAMP1	P23763	8.0	***	6843	2.8	2.4	ns	0.50	2.0	1.3	ns	0.55
CEACAM5	P06731	117.1	*	1048	4.3	3.5	ns	0.54	1.9	1.3	ns	0.54
CD177	Q8N6Q3	39.5	**	-	3.6	2.7	ns	0.58	2.1	1.6	ns	0.57
CDH17	Q12864	184.4	***	1015	3.1	2.4	ns	0.54	2.1	1.4	ns	0.58
CDH5	P33151	6.6	***	-	3.6	2.5	ns	0.60	1.9	1.4	ns	0.55

***** is the highest confidence and * is the lowest
